# Strangulated internal hernia through the lesser omentum with intestinal necrosis - a case report

**DOI:** 10.1590/S1516-31802002000300006

**Published:** 2002-05-02

**Authors:** Gustavo Gibin Duarte, Belchor Fontes, Renato Sérgio Poggetti, Marcos Roberto Loreto, Paulo Motta, Dario Birolini

**Keywords:** Strangulated internal hernia, Lesser omentum, Intestinal necrosis, Hérnia interna estrangulada, Pequeno omento, Necrose intestinal

## Abstract

**CONTEXT:**

Internal hernias account for only 0.2 to 0.9% of the cases of intestinal obstruction. They do not have specific clinical manifestations, and are usually diagnosed during laparotomy for acute intestinal obstruction. Internal hernias through the lesser omentum are extremely rare.

**CASE REPORT:**

We report here the case of a 36-year-old patient who underwent exploratory laparotomy for acute intestinal obstruction. An internal hernia through the lesser omentum was found, with a strangulated ileal segment passing through the perforation into an abscess within the lesser sac. The surgical procedures included ileal resection, primary anastomosis, abscess removal, and placement of a drain in the lesser sac. The patient was reoperated 6 days later for abdominal sepsis; a lesser sac abscess was removed and the abdominal incision was left open. The patient stayed in the Intensive Care Unit for 15 days, and eventually left the hospital on the 28 post-admission day, with complete recovery thereafter.

**CONCLUSION:**

The early diagnosis of acute intestinal obstruction and immediate indication for laparotomy is the main task of the surgeon when faced with a case of acute abdomen with a hypothesis of internal hernia, so as to minimize severe postoperative complications, as illustrated by the present case.

## RESUMO

**CONTEXTO:**

As hérnias internas do abdome correspondem apenas 0,2% a 0,9% dos casos de obstrução intestinal. Não apresentam manifestações clínicas específicas e seu diagnóstico é geralmente intra-operatório. Hérnias internas através do pequeno omento são extremamente raras.

**RELATO DE CASO:**

Relata-se aqui o caso de um paciente submetido à laparotomia exploradora por abdome agudo obstrutivo, quatro dias após o início dos sintomas. Encontrouse uma hérnia interna através do pequeno omento, com uma alça ileal estrangulada, perfurada em um abscesso na retrocavidade dos epíploos. Realizou-se ressecção da alça e anastomose íleo-ileal primária, remoção do abscesso e drenagem com dreno túbulolaminar. O paciente foi reoperado por sepse de origem abdominal seis dias depois; um abscesso retrogástrico foi removido e a incisão da parede abdominal foi deixada aberta. O paciente permaneceu 15 dias na Unidade de Terapia Intensiva e teve alta hospitalar no 28^o^ dia da internação, seguindo-se recuperação completa.

**CONCLUSÃO:**

O diagnóstico precoce de abdome agudo obstrutivo, seguido de indicação imediata de laparotomia, é a principal tarefa do cirurgião frente a um caso de abdome agudo em que a hipótese de hérnia interna está presente, visando minimizar complicações pós-operatórias graves.

## CASE REPORT

MCCG, a 36-year-old male who had previously been in good health, was admitted to the Emergency Surgery Department on May 12, 1996, complaining of having had diffuse abdominal pain, vomiting, obstipation, and abdominal distension for the preceding four days. The physical examination revealed a distended, painless abdomen and that the rectum was normal. Upright abdominal plain film showed air-fluid levels inside small bowel loops that were remarkably dis-tended. A nasogastric tube was inserted and drained out 1200 ml of brown, feculent fluid.

With a diagnosis of acute intestinal obstruction, the patient underwent an exploratory laparotomy that revealed a 90-cm ileal segment herniated through an opening in the lesser omentum. The ileal segment was strangulated, and passed through a perforation into an abscess in the lesser sac. Upon widening the hernial orifice, the ischemic bowel was removed, the abscess was evacuated, a primary anastomosis was performed and a drain was placed at the site of the abscess. Prophylactic antibiotics were administered for two days.

A condition of adynamic ileum persisted through to the 6^th^ postoperative day, and the patient was presenting systemic signs of sepsis and a purulent discharge through the abdominal wound. He therefore underwent reoperation. An abscess extending from the lesser sac to the abdominal wall incision was removed, and the abdominal wound was left open. The patient received broad-spectrum antibiotics and amphotericin B because of abscess fluid and blood cultures showing *Candida albicans*, and he stayed in the Intensive Care Unit for 15 days. Thereafter his overall condition improved, the wound healing progressed, and he was discharged 28 days after the admission. At the follow-up two months later he was completely recovered.

## DISCUSSION

Internal hernias result from the protrusion of one or more abdominal viscera through an intra-peritoneal opening, with the herniated viscera remaining inside the abdominal cavity. These openings can be normal (e.g. Winslow's foramen), paranormal (e.g. paraduodenal fossa, ileocecal fossa, supravesical fossa), and also abnormal anatomical entities (e.g. transomental defects).^1,2^ Predisposing factors for transomental hernias include congenital anatomic defects of the liver, lesser sac, mesentery, as well as the presence of adhesions or increased intra-abdominal pressure.^3,4^ Abnormal transomental openings are usually congenital, and rarely traumatic or iatrogenic.^5^ Internal hernias are infrequent, accounting for 0.2 to 0.9% of the cases of intestinal obstruction,^6^ and lead to 0.5 to 4.1% of the cases of acute intestinal obstruction caused by hernias.^7-9^ Transomental hernias through the greater or lesser omentum are even rarer, representing 1 to 4% of all internal hernias,^5^ with hernias occurring through the lesser omentum being extremely rare.^5,10-12^

The literature on internal hernias,^4,8,9,13-17^ which includes over 50 articles published during the last decade, emphasizes that a hypothesis of internal hernia should be considered for patients with signs and symptoms of intestinal obstruction, particularly in the absence of inflammatory intestinal diseases, external hernia, or previous laparotomy. The extreme difficulty in making diagnoses of internal hernia, because of the lack of specific signs and symptoms, is also emphasized.^15^ On the other hand, internal hernias are invariably manifested as acute intestinal obstruction that requires early diagnosis and immediate surgery. In addition, the value of modern diagnostic imaging tools in the specific diagnosis of internal hernia, particularly computed tomography, is in practice limited to cases of partial obstruction in which surgical management is usually not required. As a result, internal hernias are usually diagnosed during laparotomy for acute intestinal obstruction.^4,13,14,17^

The overall management of acute intestinal obstruction requires appropriate initial resuscitation and nasogastric tube decompression, followed by immediate laparotomy. A median laparotomy incision, followed by upper or lower extension when needed, is usually adequate for accessing the unpredictable site of the obstructive process, for any procedure required. However, access to hernias of the retrogastric cavity may require a wide opening into either the lesser omentum or the gastrocolic ligament.^5^ A finding of herniation through an intra-peritoneal orifice makes a diagnosis of internal hernia manifest. Next, a careful examination of the abdominal cavity is performed in order to identify the organs/structures involved in the obstruction, and to check for the presence of ischemia, necrosis, perforation and contamination.

The surgical maneuvers for the management of internal hernias include reduction of the herniated structures, resection of ischemic intestinal segments, and closure of the hernial orifice.^9^ Reduction of the herniated intestinal segment may be simple, by means of delicate traction, or difficult requiring dilatation or widening of the hernial orifice as well as opening of the hernial sac. Dilatation is performed by delicate digital maneuvers, avoiding herniated loops or blood vessel lesions. Widening of the orifice has the risk of vascular lesion, which can be avoided through knowledge of the anatomy of the peritoneal fossae and regional vessels. Resection of the hernial sac is almost always unnecessary.^4,5^ Mild ischemia of the herniated intestinal loop frequently is reverted a few minutes after the loop is released. In the presence of necrosis, perforation or irreversible ischemia, intestinal resection is performed.^9^ Primary intestinal anastomosis is almost always indicated, with exteriorization reserved for exceptional situations. Closure of the hernial orifice is generally indicated for the prevention of the recurrence of hernias through abnormal orifices (except for complex cases like the one here reported). Closure of normal orifices is controversial: for example, closure of Winslow's foramen has a potential risk for portal thrombosis.^5^

**Figure:**
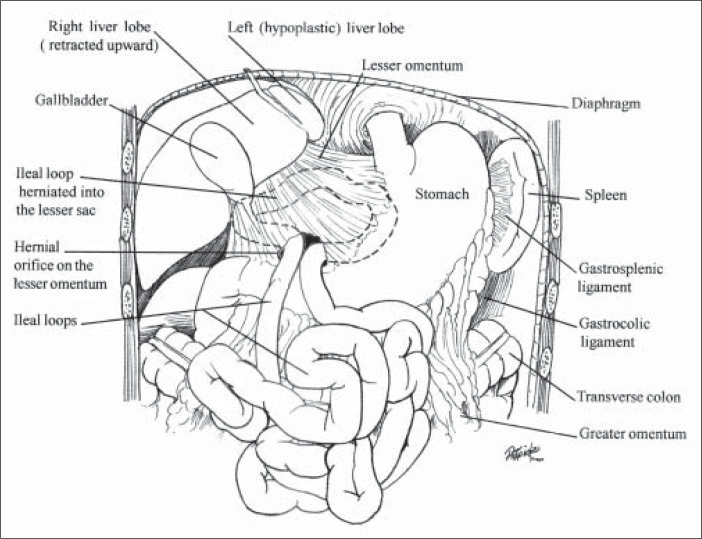


In conclusion, when faced with a clinical condition of acute intestinal obstruction, as in the case here reported, in which there is a hypothesis of internal hernia, the surgeon's main task is to provide early exclusion or confirmation of the diagnosis of acute intestinal obstruction. In the latter case, immediate laparotomy must be indicated, with no priority for the specific diagnosis of the cause of the obstruction. This policy has the aim of reducing the risk of intestinal ischemia, necrosis and perforation, and decreasing postoperative morbidity and mortality.
